# A Four Green TM/Red TE Demultiplexer Based on Multi Slot-Waveguide Structures

**DOI:** 10.3390/ma13143219

**Published:** 2020-07-20

**Authors:** Dror Malka

**Affiliations:** Faculty of Engineering, Holon Institute of Technology (HIT), Holon 5810201, Israel; drorm@hit.ac.il

**Keywords:** demultiplexer, FV-BPM, slot waveguide, VLC

## Abstract

A four green transverse magnetic (TM)/red transverse electric (TE) light wavelength demultiplexer device, based on multi slot-waveguide (SW) structures is demonstrated. The device aims to demultiplex wavelengths in the green/red light range with wavelengths of 530, 540, 550, and 560 nm; 630, 640, 650, and 660 nm. This means that the device functions as a 1 × 4 demultiplexer for each polarization mode (TE/TM). The controlling of the light switching between two closer segment SWs under the TM/TE polarization mode was studied by designing a suitable SW structure and setting the right segment length to fit the coupling lengths of the operating wavelengths. The device is composed of six-segment SW units and six S-bends (SB) SW. The key SW and SB parameters were optimized and determined by a full vectorial beam propagation method (FV-BPM). Results show power losses better than 0.138 dB, crosstalk better than −21.14 dB, and an optical spectrum smaller than 9.39 nm, with an overall compact size of 104.5 µm. The device can be integrated in wavelength division multiplexing (WDM) for increasing data bit rate in a visible light communication (VLC) system.

## 1. Introduction

The fundamental of wavelength division multiplexing (WDM) lies in the ability to send different data types over waveguide networks in the form of light [1]. By allowing different light channels, each with a unique wavelength, to be sent simultaneously over a waveguide network a single virtual waveguide network is created [2].

WDM devices have been demonstrated [3] using waveguide technology such as echelle grating [4] and angled multi-mode interference (MMI) [5].

Recently, visible light communication (VLC) become more popular due to the rise of wireless data communication in the world [6,7]. The advantages of using VLC have been studied and the main advantages are no health risks to human beings, can be used for both illumination and communication, and low power consumption [6,8].

However, the main challenges of using the VLC system are the high path loss due to the reasonably high frequency [6] and to obtain a high data communication bit rate. The solution to overcoming these problems is to use a very low-loss waveguide technology such as slot-waveguide (SW).

SW has several properties that enable the ability to transmit light without confinement losses [9] and to use any low-loss semiconductor materials.

An SW structure is a complex consisting of two layers of high-index materials surrounding a layer of low-index material. This nanoscale structure enables the total internal reflection mechanism to guide and confine the light under both polarization mode transverse magnetic (TM) and transverse electric (TE) mode inside the SW region [9]. The light is strongly confined in the slot (low-index) area under TM polarization mode and for TE polarization mode the light is strongly confined in the high-index areas. Studies have been done on SW devices [10,11,12,13] including SW devices that work in the visible light spectrum. SW can be designed to work in the visible range under TM and TE polarization mode [7,14]. Coupling mode theory between two closer waveguides has been well studied, and works have been demonstrated [15,16,17].

Former works, which studied demultiplexers based on SW in MMI structures operating within the visible spectrum and in the C band, show a good performance and an overall size device as long as 700 µm and up to several millimeters [2,18].

However, this technology cannot be implemented to design a demultiplexer device that can work on both polarization mode TM and TE. This is because the MMI coupler geometrical parameters have a polarization dependency which limits it to function only for the TE or TM polarization mode. To overcome this problem, I proposed to work on multi SW technology to utilize the SW structure that has the pentanal in optimal design to work on both polarization modes TM and TE.

The novelty of a design demultiplexer using a multi SW structure is the ability to work on both polarization modes TM and TE. This ability can be utilized for increasing the number of wavelengths that can be demultiplexed without changing the device dimensions.

Another ability is to obtain a low coupling length while staying on low sensitivity to the variation of the operating wavelengths and in other words, the overall device size not being sensitive to the variation of the operating wavelengths. These abilities can lead to an ultra-compact size that can be used for obtaining better device performances.

In this paper, we present a study of the coupling mechanism between six parallels segment SW units and the oscillation characteristic of the green/red light between them to realize an efficient four channel demultiplexer device.

The demultiplexer operation is based on controlling the light coupling between two closer segment SW units. This light controlling can be done by designing an SW structure that can guide light in both polarization modes TE and TM and optimize the segment SW length that enables a full energy transfer between two closer segment SW units under both polarization modes TE and TM.

Gallium nitride (GaN) and silica (slot) were found to be excellent low-loss materials (1–2.5 dB/cm) [7] for the SW structure. Coupling light into the device can be done using a mode field diameter converter implemented as an input taper [19].

The demultiplexer was designed to divide four green/red wavelengths with an accurate spacing of 10 nm. A full vectorial beam propagation method (FV-BPM), under a TM/TE polarization mode, was used to analyze and optimize the key geometrical parameters of the six segment SW and SB units.

## 2. Theory and Design

**Figure 1:**
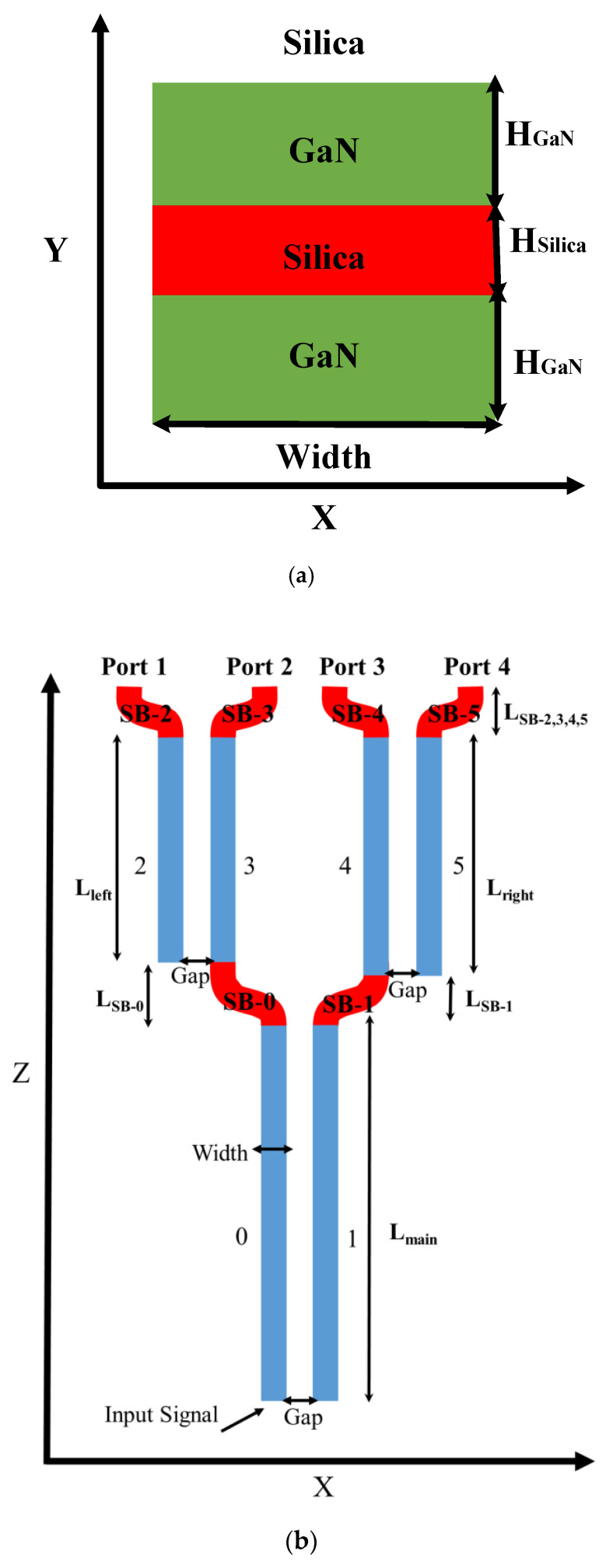
A schematic sketch of the 1 × 4 red/green wavelength demultiplexer. (**a**) SW structure at the x–y plane. (**b**) The full structure design of the six SWs at the x–z plane.

Figure 1a shows a cross-section of the SW unit at the x–y plane. The two layers (H_GaN_) of GaN (green color) sandwich a middle slot layer (H_Silica_) of silica (red color) and the cladding (white color) is composed of silica. The width of the SW unit described as width, is shown in Figure 1a,b. In this case, the SW unit is set to be in a horizontal structure as can be seen in Figure 1a. The reason is that the device functions as a wavelength splitter which is much more efficient as a horizontal structure [20]. A vertical SW unit structure can be described by rotating Figure 1a 90 degrees clockwise. From the fabrication view, the vertical slot involves etching in a very narrow area that can lead to large roughness in the vertical interfaces which limit the device performances [21].

Figure 1b shows a full schematic scheme of the six segment SW units (light-blue color) and the six SB (red color) SWs of the proposed demultiplexer at the x–z plane. The two main segment SW (SW-0 and SW-1) have a length defined as L_main_. The left pair of segment SW (SW-2 & SW-3) has a length defined as L_left_, and the right pair (SW-4 and SW-5) have a length defined as L_right_. Regarding the six SB SWs, the lengths of SB-0, SB-1, SB-2, SB-3, SB-4 and SB-5 are defined as LSB-0, LSB-1, LSB-2, LSB-3, LSB-4 and LSB-5, respectively. Where the length of SB-2,3,4,5 is equal. The size of the gap between two segment SW units is defined as Gap and it is constant through all the structure.

The proposed SW unit can be fabricated using the E-beam lithography technique [22]. This fabrication technique can be used in a similar way to fabricated GaN waveguide structures such as rib waveguide based on Ga_2_O_3_ [23], Al_2_0_3_ [24], and silicon on insulator substrate [25].

The coupling length (L_Coupling_) between two segment SW units is dependent on the key geometrical parameters (Gap, Width), polarization mode and the operating wavelength. The coupling length under the TM/TE polarization mode can be found by using FV-BPM simulation that shows the transfer energy between two closer segment SW units. The coupling length is given by:

(1) LCoupling=λ2[ne(λ,Gap,width,polarization)−no(λ,Gap,width,polarization)]

where n_e_ and n_o_ are the effective refractive indices of the even and odd supermodes, respectively.

The conditions for separating four different wavelengths [26] using the six segment SW units are given by:

(2) $$\begin{array}{l} L_{\mathrm{main}}\approx p_{1}L_{\mathrm{Coupling}}^{\lambda_{1},\lambda_{3}}=(p_{1}+q_{1})L_{\mathrm{Coupling}}^{\lambda_{2},\lambda_{4}}\\ L_{\mathrm{left}}\approx p_{2}L_{\mathrm{Coupling}}^{\lambda_{2}}=(p_{2}+q_{2})L_{\mathrm{Coupling}}^{\lambda_{4}}\\ L_{\mathrm{right}}\approx p_{3}L_{\mathrm{Coupling}}^{\lambda_{1}}=(p_{3}+q_{3})L_{\mathrm{Coupling}}^{\lambda_{3}} \end{array}$$

Here L_main_ is the length of the main segment SW units, L_left_ is the length of the left segment SW units, L_right_ is the length of the right segment SW units, p_1/2/3_ is a natural number, and q_1/2/3_ is an odd number.

The device design is further constrained by the requirement to make the demultiplexer polarization independent, such that both polarizations mode TE and TM of a given operating wavelength exit the same port together. In our case, the device operates in both polarization modes TE and TM. To split two wavelengths without splitting the polarization, it must satisfy the polarization coupling length conditions:

(3) $$\begin{array}{ll} L & =pL^{\mathrm{TE}}{}_{\mathrm{Coupling}}(\lambda_{1})=\left(p+q\right)L^{\mathrm{TM}}{}_{\mathrm{Coupling}}(\lambda_{1})\\ & =(p+d)L^{\mathrm{TE}}{}_{\mathrm{Coupling}}(\lambda_{2})=\left(p+d+q^{\prime }\right)L^{\mathrm{TM}}{}_{\mathrm{Coupling}}(\lambda_{2}) \end{array}$$

where L is the segment SW length, d is an odd number, and q and q’ are even numbers. This strong polarization and wavelength dependence can be found using SW technology. Thus, by using Equation (3) into Equation (2) L_main,_ L_right,_ and L_left_ can be defined for both polarization mode TE and TM. The conditions for separating four different wavelengths under both polarization mode TE and TM using the six-segment SW units are given by:

(4) $$\begin{array}{ll} L_{\mathrm{main}} & \approx p_{1}L_{\mathrm{Coupling}}^{\mathrm{TE}}(\lambda_{1},\lambda_{3})=(p_{1}+q_{1})L_{\mathrm{Coupling}}^{\mathrm{TM}}(\lambda_{1},\lambda_{3})\\ & =(p_{1}+d)L_{\mathrm{Coupling}}^{\mathrm{TE}}(\lambda_{2},\lambda_{4})=(p_{1}+d+q_{1}^{\prime })L_{\mathrm{Coupling}}^{\mathrm{TM}}(\lambda_{2},\lambda_{4})\\ L_{\mathrm{left}} & \approx p_{2}L_{\mathrm{Coupling}}^{\mathrm{TE}}(\lambda_{2})=(p_{2}+q_{2})L_{\mathrm{Coupling}}^{\mathrm{TM}}(\lambda_{2})\\ & =(p_{2}+d_{2})L_{\mathrm{Coupling}}^{\mathrm{TE}}(\lambda_{4})=(p_{2}+d_{2}+q_{2}^{\prime })L_{\mathrm{Coupling}}^{\mathrm{TM}}(\lambda_{4})\\ L_{\mathrm{right}} & \approx p_{3}L_{\mathrm{Coupling}}^{\mathrm{TE}}(\lambda_{1})=(p_{3}+q_{3})L_{\mathrm{Coupling}}^{\mathrm{TM}}(\lambda_{1})\\ & =(p_{3}+d_{3})L_{\mathrm{Coupling}}^{\mathrm{TE}}(\lambda_{3})=(p_{3}+d_{3}+q_{3}^{\prime })L_{\mathrm{Coupling}}^{\mathrm{TM}}(\lambda_{3}) \end{array}$$

To enable the conditions in Equation (3) the SW structure has to be guided and strongly confine the light for both polarization mode TE and TM.

By assuming a full energy transfer between SW-0 and SW-1, SW-2 and SW-3 and SW-4 and SW-5, the classic coupling equations can be described as follows:

(5) $$\begin{array}{l} \frac{da_{0}}{\mathrm{dz}}+j\beta a_{0}=-ja_{1}\kappa_{1},\;0<z<L_{\mathrm{main}},\;\mathrm{For}:\lambda_{1/2/3/4}\\ \frac{da_{1}}{\mathrm{dz}}+j\beta a_{1}=-ja_{0}\kappa_{1},\;0<z<L_{\mathrm{main}},\;\mathrm{For}:\lambda_{1/2/3/4}\\ \frac{da_{2}}{\mathrm{dz}}+j\beta a_{2}=-ja_{3}\kappa_{2},\;L_{\mathrm{main}}+L_{\mathrm{SB}-0}<z<L_{\mathrm{total}},\;\mathrm{For}:\lambda_{2/4}\\ \frac{da_{3}}{\mathrm{dz}}+j\beta a_{3}=-ja_{2}\kappa_{2},\;L_{\mathrm{main}}+L_{\mathrm{SB}-0}<z<L_{\mathrm{total}},\;\mathrm{For}:\lambda_{2/4}\\ \frac{da_{4}}{\mathrm{dz}}+j\beta a_{4}=-ja_{5}\kappa_{3},\;L_{\mathrm{main}}+L_{\mathrm{SB}-1}<z<L_{\mathrm{total}},\;\mathrm{For}:\lambda_{1/3}\\ \frac{da_{5}}{\mathrm{dz}}+j\beta a_{5}=-ja_{4}\kappa_{3},\;L_{\mathrm{main}}+L_{SB-1}<z<L_{total},\;\mathrm{For}:\lambda_{1/3} \end{array}$$

where a_0_, a_1_, a_2_, a_3_, a_4_, and a_5_ are the amplitudes of the mode, κ_1_, κ_2_ and κ_3_ are the coupling coefficient between the main pair of SWs, left pair of SWs and right pair of SWs respectably, β is the propagation of the light, j is the imaginary number, dz is the differential in the z–axis, and L_total_ is the overall length size of the device.

The boundary conditions, in our case, are given by:

(6) $$\begin{array}{l} a_{0}(z=0)=1,\;\mathrm{For}:\lambda_{1/2/3/4}\\ a_{1}(z=0)=0,\;\mathrm{For}:\lambda_{1/2/3/4}\\ a_{2}(z=L_{\mathrm{main}}+L_{\mathrm{SB}-0})=0,\;\mathrm{For}:\lambda_{2/4}\\ a_{3}(z=L_{\mathrm{main}}+L_{\mathrm{SB}-0})=1,\;\mathrm{For}:\lambda_{2/4}\\ a_{4}(z=L_{\mathrm{main}}+L_{\mathrm{SB}-1})=1,\;\mathrm{For}:\lambda_{1/3}\\ a_{5}(z=L_{\mathrm{main}}+L_{\mathrm{SB}-1})=0,\;\mathrm{For}:\lambda_{1/3} \end{array}$$

The solution for the classical coupled equation can be given by:

(7) $$a_{i}=A_{n}\exp(-jz(\beta +\mu ))$$

where µ is an eigenvalue. Also, optimizations on the lengths of L_main_, L_right_, and L_left_ were adjusted due to the coupling contribution of SB-0/1, SB-4/5, and SB-2/3 respectively, to obtain a better performance.

For analyzing the performances of our presented multi SW wavelength demultiplexer, crosstalk (Equation (8)) [27] and loss (Equation (9)) were calculated to observe the ratio between a desirable and undesirable operated wavelength in a given port and the power losses respectively.

(8) $${C.T}_{n}=\frac{1}{3}\sum_{m=1}^{4}10\log\left(\frac{P_{m}}{P_{n}}\right)$$

where P_n_ is the power transmission for the suitable n-port, and P_m_ is the interference power transmission from the other ports.

The insertion loss is given by:

(9) $${\mathrm{Loss}}_{\mathrm{dB}}=-10\log\left(\frac{P_{\mathrm{out}}}{P_{\mathrm{in}}}\right)$$

where P_out_ is the power at the output port, and P_in_ is the power in the input SW (SW-0).

For the green/red operating wavelengths, the refractive index values for the SW structure are shown in Table 1.

## 3. Simulation Results

**Figure 2:**
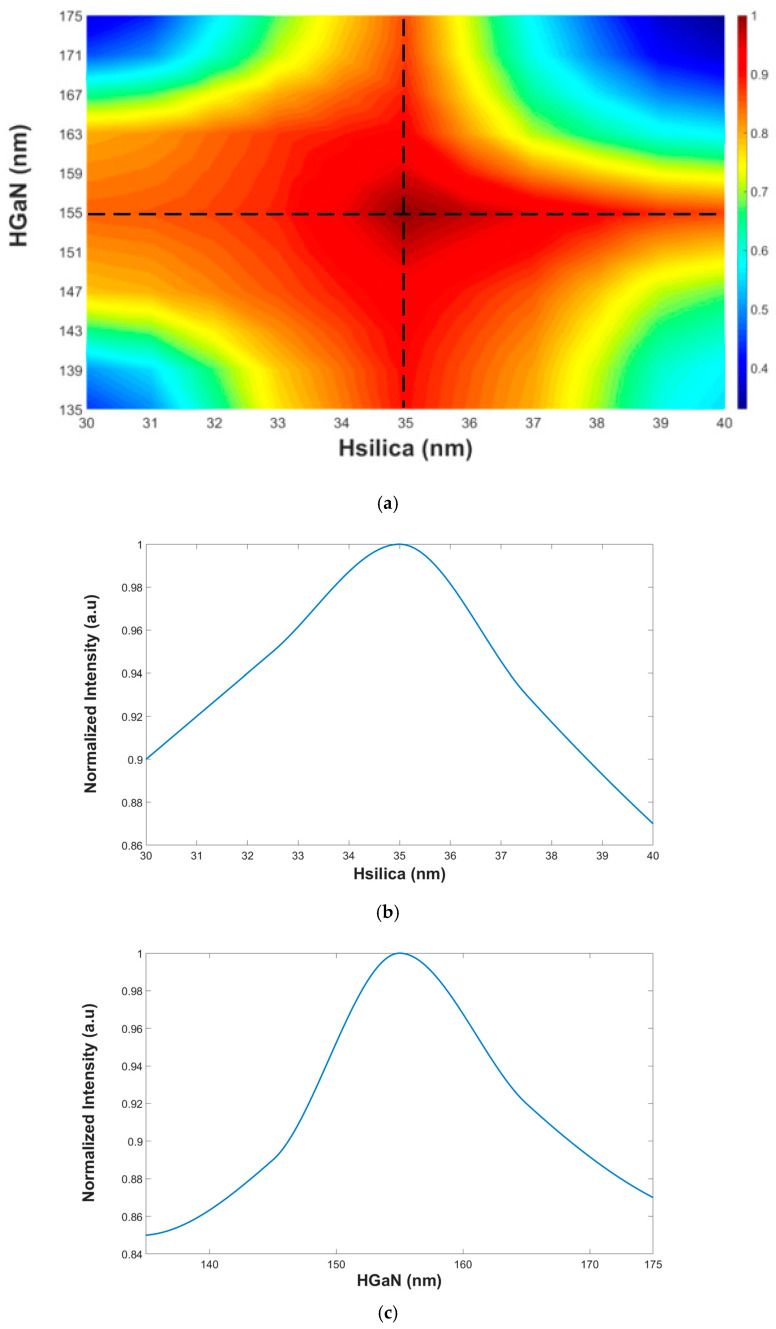
The normalized intensity as a function of the SW layer thickness. (**a**) 2D normalized intensity colormap view. (**b**) Silica layer thickness. (**c**) GaN layer thickness.

**Figure 3:**
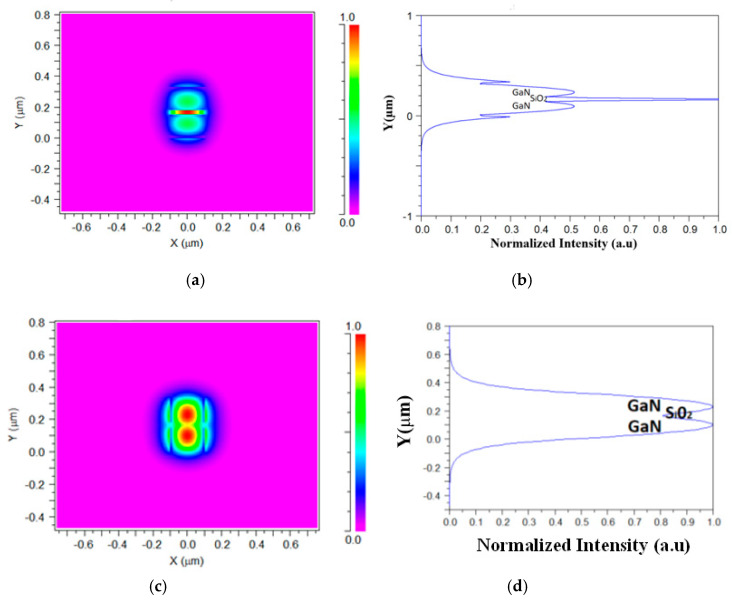
Fundamental mode profile inside the SW at the x–y plane (**a**) TM mode (E_y_). (**b**) A vertical cut of Ey at x = 0 nm. (**c**) TE mode (E_X_). (**d**) A vertical cut of E_X_ at x = 0 nm.

The simulations were done using an FVBPM based on Rsoft-CAD numerical software. Also, Matlab script codes were used to analyze the simulation calculations. From these simulations, the optimal geometrical parameters of the SW structure were set to be: H_slot_ = 35 nm, H_slab_ = 155 nm, and width = 205 nm. These values were used to obtain a strong intensity light confinement inside the silica/GaN area/areas under TM/TE polarization mode. Figure 2a shows the 2D colormap normalized intensity view as a function of both silica and GaN thickness layer under an operating wavelength of 640 nm. Figure 2b shows the normalized intensity as a function of the silica layer thickness (H_Silica_) that represents the cut section at H_GaN_ = 155 nm in Figure 2a (black dashed line). Figure 2c shows the normalized intensity as a function of the GaN layer thickness (H_GaN_) that represents the cut section at H_Silica_ = 35 nm in Figure 2a (black dashed line). The normalized intensity represents the level value of the light confinement inside the SW. For TM mode the light level is high in the slot area (red color in Figure 3a) and for TE mode the light level is high in the GaN areas (red color in Figure 3c). It is important to indicate that the same results were obtained for the other operating wavelengths. From Figure 2a,b it can be seen that the optimal values of the SW layers are 35 nm (H_Silica_) and 155 nm (H_GaN_). From Figure 2c the tolerance ranges for silica and GaN thickness layer that ensures a strong light confinement (above 90% of the normalized intensity) inside the SW under TE and TM mode can be found and their values are 33.5–37.5 nm and 144–163 nm.

Figure 3a shows the TM fundamental mode profile inside the SW structure at the x–y plane, for a wavelength of 530 nm. It can be observed that the power is highly confined inside the silica layer (red color). Figure 3b shows the vertical cut of Ey (Figure 3a) at x = 0 nm and the light level intensity for each layer. The same mode profile was obtained for the other TE operated wavelengths (540, 550, and 560 nm). Figure 3c shows the TE fundamental mode profile inside the SW structure at the x–y plane, for a wavelength of 630 nm. It can be seen that the power is highly confined inside the GaN layers (red color) and a wide range of tolerance along the y–axis (around 33 nm) is visible. Figure 3d shows the vertical cut of Ex (Figure 3c) at x = 0 nm and the light level intensity for each layer. The same mode profile was obtained for the other TM operated wavelengths (640, 650, and 660 nm).

**Figure 4:**
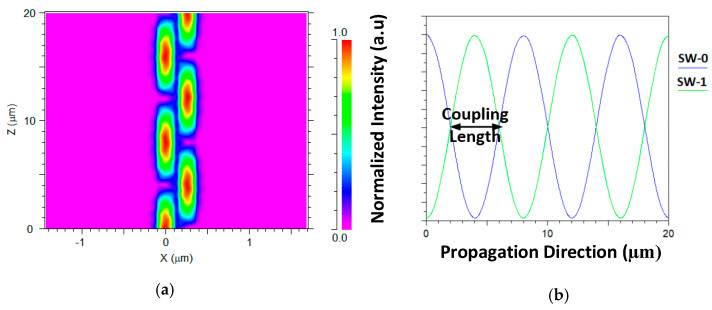
Light coupling between two closer segment SW units under TE polarization mode at 630 nm (**a**). Light propagation over z–axis (**b**). Energy transfer between the two segment SW channels.

Figure 4a shows the light coupling between two closer segment SW units (0 and 1) over the z–axis from 0 to 20 µm under the TE polarization mode at 630 nm wavelength. Figure 4b shows the full transfer energy (around 100% from the normalized intensity) between SW unit 0 and SW unit 1 and the coupling length value. Using this numerical technique, the other coupling length values which are suitable for the operating wavelengths can be found as shown in Table 2.

**Figure 5:**
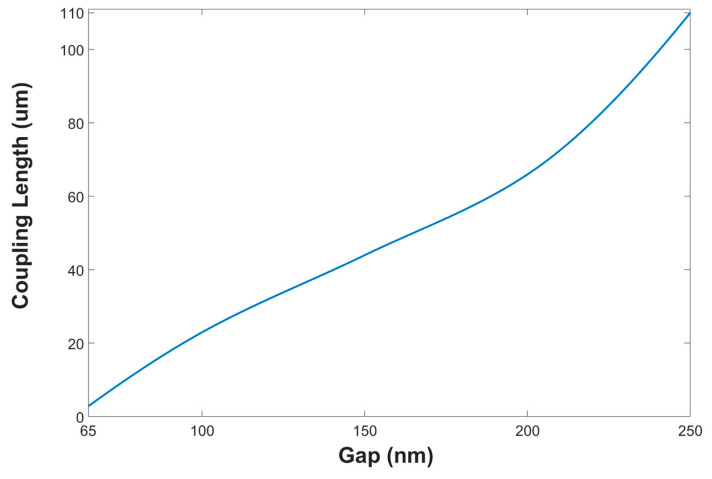
Coupling length as a function of the Gap between two closer SW units with 630 nm wavelength.

Figure 5 shows the coupling length between two closer segment SW units as the function of the gap between them with a wavelength of 630 nm in TE polarization mode. The same physical behavior was obtained for the other operating wavelengths under TE and TM polarization mode.

In this figure, it is clear that the optimal gap value of 65 nm leads to a compact device size.

It is important to emphasize that the geometry of the device, was optimized for the green and the red operated wavelengths. After analyzing the results shown in Figure 4 and Figure 5, Table 2, and solving Equations (1)–(4), the SW geometrical parameters were optimized to be, 62.72 µm, 31.7 µm, and 30.2 µm, for L_main_, L_right_, and L_left_ respectively. These yield an overall device length of 104.5 µm, including the benefits of the six SBs, with lengths of 9.25 µm, 7.75 µm, and 2.5 µm for LSB-0, LSB-1, and LSB-2,3,4,5 respectively.

**Figure 6:**
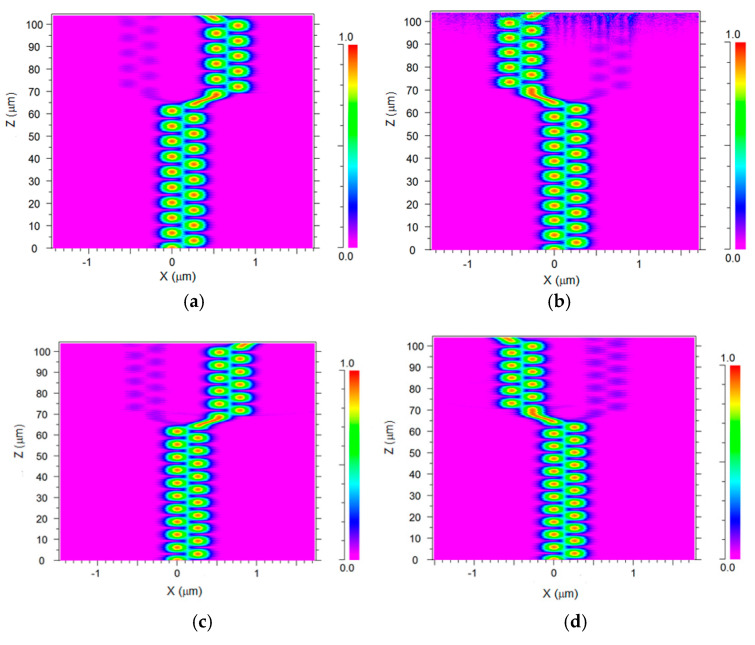
Intensity light profile of the 1 × 4 TM polarization wavelength demultiplexer at the x–z plane. (**a**) 530 nm (port 3). (**b**) 540 nm (port 2). (**c**) 550 nm (port 3). (**d**) 560nm (port 1).

**Figure 7:**
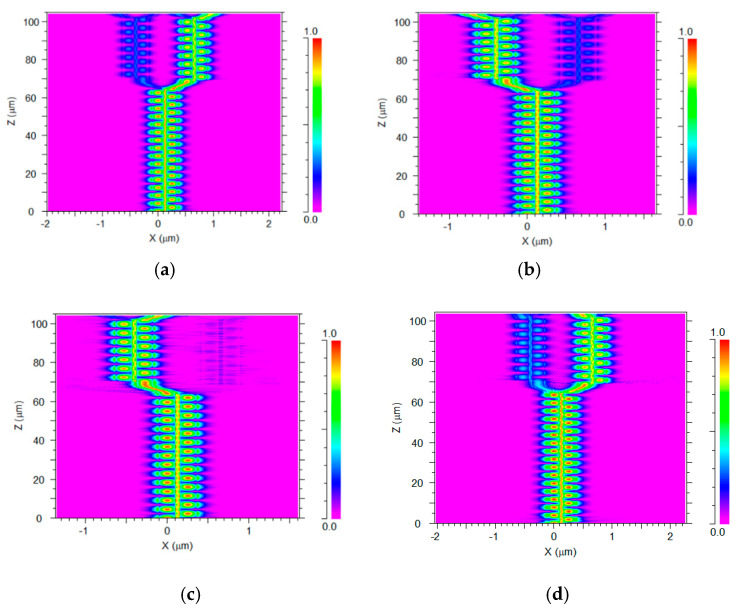
Intensity light profile of the 1 × 4 TE polarization wavelength demultiplexer at the x–z plane. (**a**) 630 nm (port 4). (**b**) 640 nm (port 1). (**c**) 650 nm (port 2). (**d**) 660 nm (port 3).

Figure 6a–d show the green light propagation for each operating wavelength through the six SW units. Figure 7a–d show the red light propagation for each operating wavelength through the six SW units.

For each operating wavelength, the light is propagated from SW-0, then the light is coupled between SW-0 and SW-1 (the two first equations of the classic coupling Equations (5)), over the z–axis. In these simulations, the light source was set to be the fundamental mode for each operated wavelength under polarization mode TM (530, 540, 550, and 560 nm) and TE (630, 640, 650, and 660 nm).

In Figure 6a and Figure 7d light with a wavelength of 530 or 660 nm propagates from SW-1 to SB-1 and is guided to SW-4. Then the light is coupled and oscillates between SW-4 and SW-5 (the last two equations of the classic coupling Equation (5)) until propagate guiding from SB-4 (Port-3). In Figure 6b and Figure 7c light with a wavelength of 540 or 650 nm propagates from SW-0 to SB-0 and is guided to SW-3. Then the light is coupled and oscillates between SW-2 and SW-3 (the third and fourth equations of the classic coupling Equation (5)) until it propagates from SB-3 (Port-2). In Figure 6c and Figure 7a light with a wavelength of 550 or 630 nm propagates from SW-1 to SB-1 and is guided to SW-4. Then the light is coupled and oscillates between SW-4 and SW-5 until it propagates from SB-5 (Port-4). In Figure 6d and Figure 7b light with a wavelength of 530 or 640 nm propagates from SW-0 to SB-0 and is guided to SW-3. Then the light is coupled and oscillates between SW-2 and SW-3 until it propagates from SB-2 (Port-1). The light coupling behavior of the light, as shown in Figure 6a–d and Figure 7a–d, reflects the classical coupled mode theory shown in Equations (5) and (6). The controlling of the light coupling between two closer segment SW units can be observed in these figures. For example, by viewing Figure 6a,c and Figure 7c,d it can be seen that there is around 19–20, 22–23, 30–31, and 33–34 light transfer between SW-0 and SW-1 that is suitable to the conditions in Equation (4). From these figures, the coupling losses between the closer SW units can be seen (blue color). In our case, each operated wavelength (red or green) guided light over an optical path that included two coupling areas. The energy transfer in these areas was not fully 100% (around 96.9–99.5%) which leads to very low coupling losses between the SW units as can be seen in Figure 6a–d and Figure 7a–d. Also, the residual losses are very low due to the use of output ports SB SW with a large radius of 25.06 µm.

**Figure 8:**
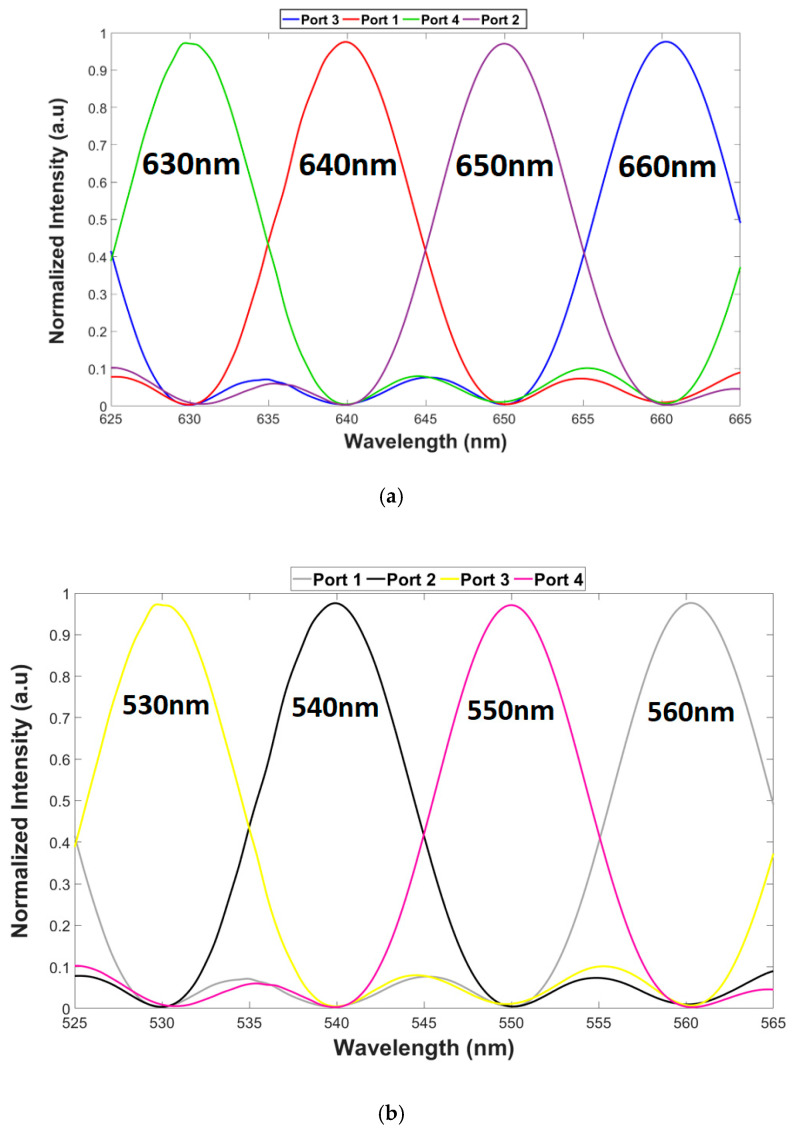
Visible light spectrum. (**a**). Red wavelength range under TE mode. (**b**) Green wavelength range under TM mode.

Figure 8a,b shows the spectral transmission over the green and red wavelength spectrum range (525–565, 625–665 nm). It can be noticed, that an accurate spacing of 10 nm was achieved between the four red/green wavelengths. Also, the values from Table 3, crosstalk, loss, and bandwidth, were found using Figure 8a,b and Equations (8) and (9). It can be seen that each wavelength has a different loss. This is because the coupling and the residual losses are different for each operating wavelength as shown in Table 3.

**Figure 9:**
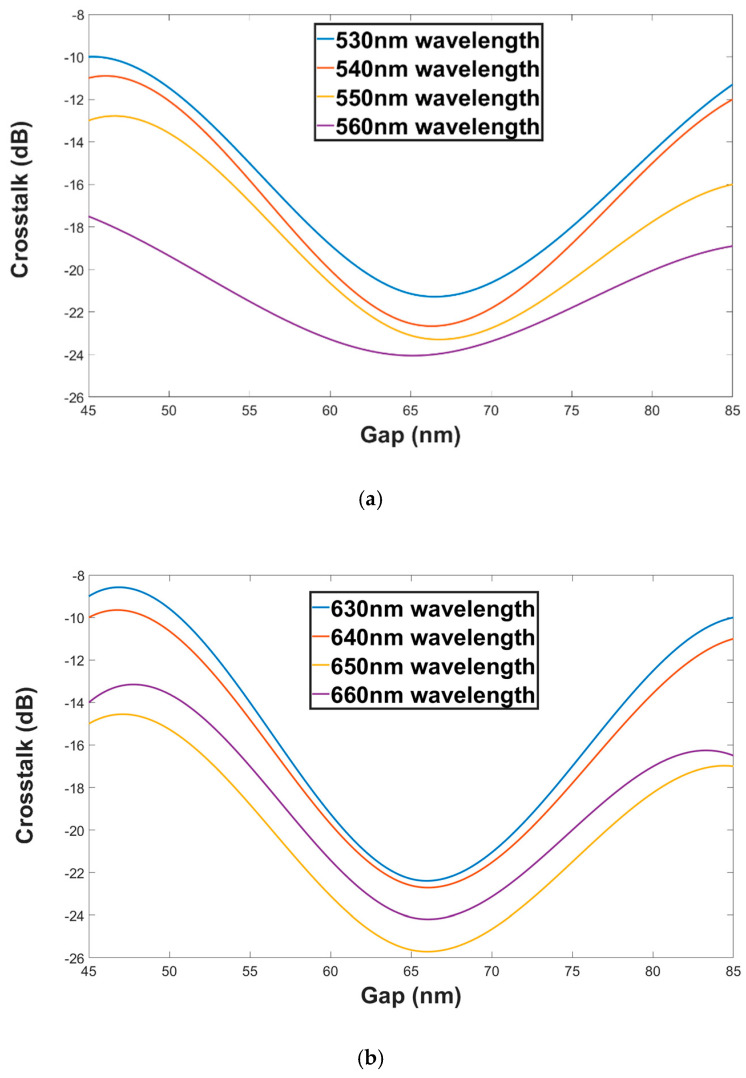
Crosstalk as a function of the Gap (**a**). Green wavelengths (TM mode). (**b**) Red wavelengths (TE mode).

Figure 9a,b show the crosstalk as a function of the Gap for both polarization modes TM (green wavelengths) and TE (red wavelengths). From these figures, it can be seen that the crosstalk range values for green and red wavelengths under ±20 nm shift from the Gap optimal value are −10.1 to −24.05 dB and −9.1 to −25.63 dB, respectively.

**Figure 10:**
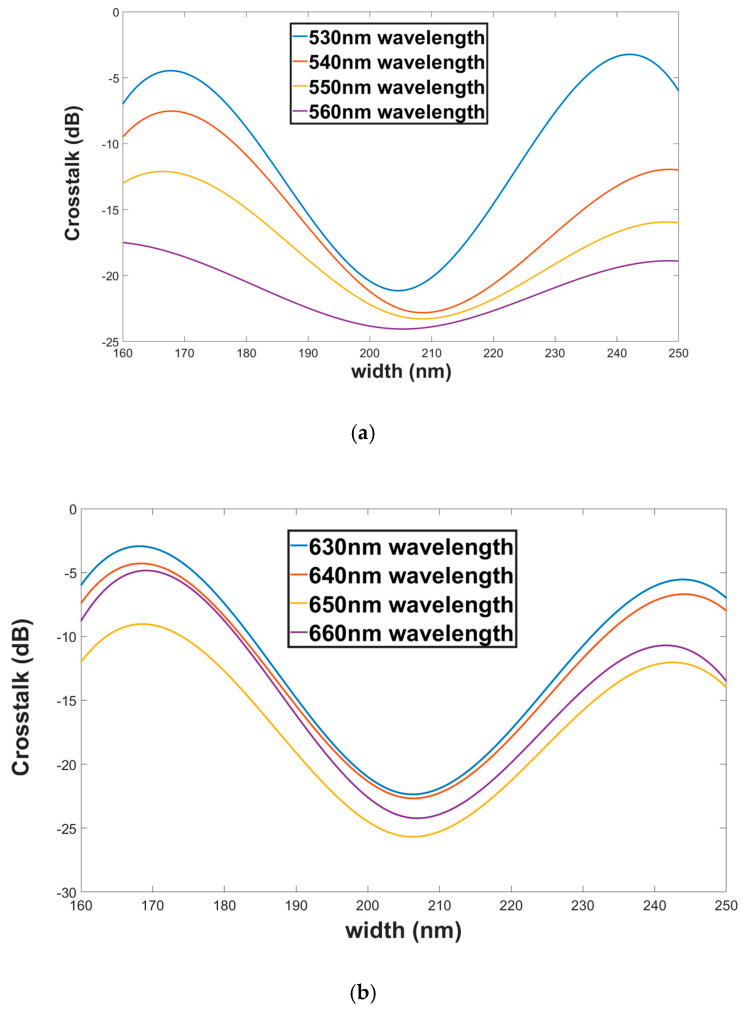
Crosstalk as a function of the SW width (**a**). Green wavelengths (TM mode). (**b**) Red wavelengths (TE mode).

Figure 10a,b show the crosstalk as a function of the SW width for both polarization modes TM (green wavelengths) and TE (red wavelengths). From these figures, it can be seen that the crosstalk range values for green and red wavelengths under ±45 nm shift from the SW width optimal value are −7.09 to −24.05 dB and −6.18 to −25.63 dB, respectively.

**Figure 11:**
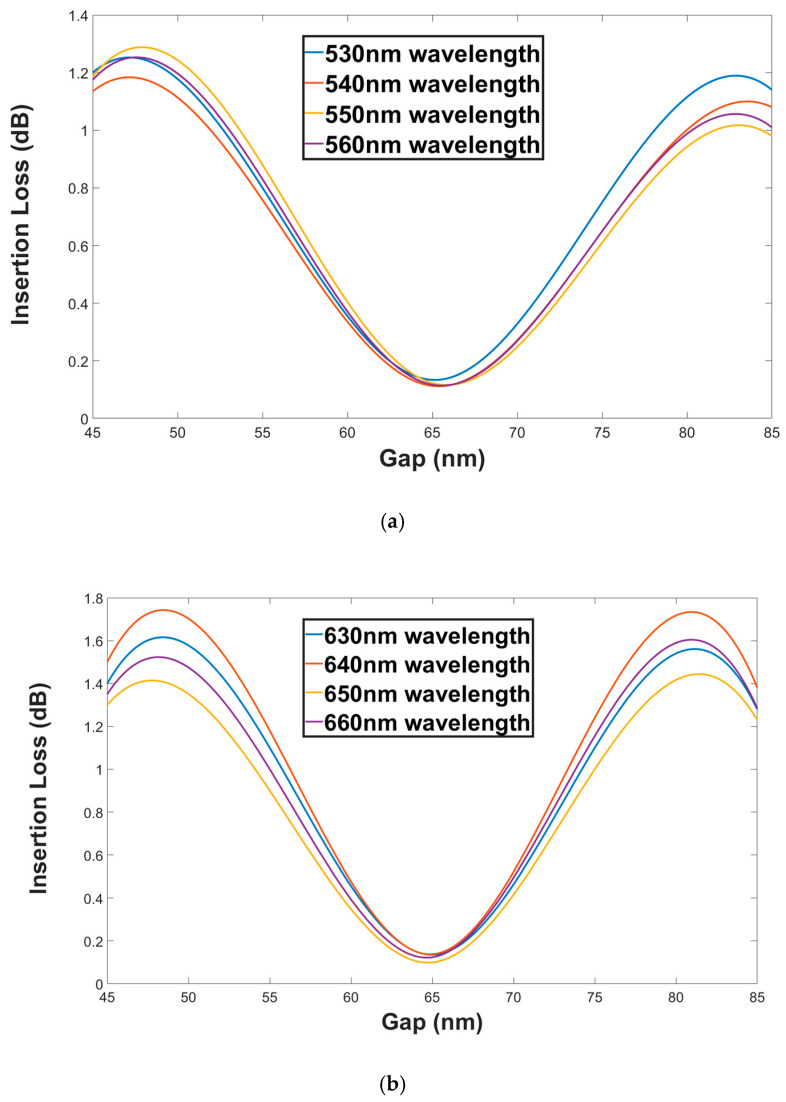
Insertion loss as a function of the Gap (**a**). Green wavelengths (TM mode). (**b**) Red wavelengths (TE mode).

Figure 11a,b shows the insertion loss as a function of the Gap for both polarization mode TM (green wavelengths) and TE (red wavelengths). From these figures, it can be seen that the insertion loss range values for green and red wavelengths under ±20 nm shift from the Gap optimal value are 1.26 to 0.113 dB and 1.66 to 0.1 dB, respectively.

**Figure 12:**
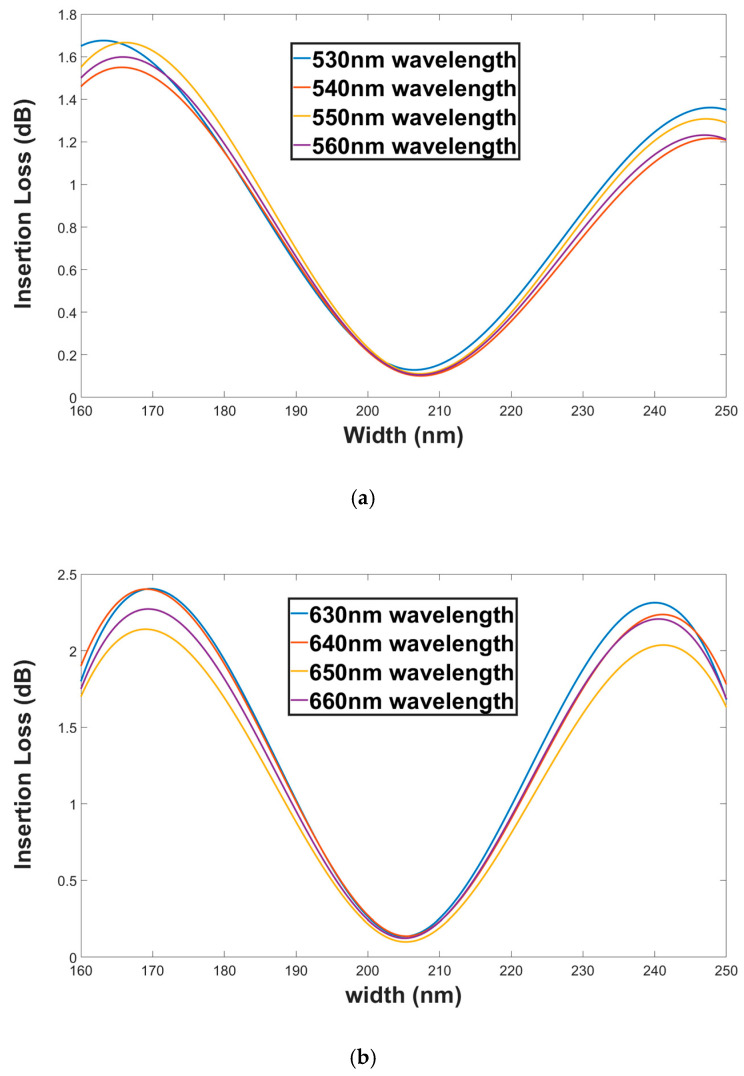
Insertion loss as a function of the SW width (**a**). Green wavelengths (TM mode). (**b**) Red wavelengths (TE mode).

Figure 12a,b shows the insertion loss as a function of the SW width for both polarization modes TM (green wavelengths) and TE (red wavelengths). From these figures, it can be seen that the insertion loss range values for green and red wavelengths under ±45 nm shift from the SW width optimal value are 1.63 to 0.113 dB and 2.28 to 0.1 dB, respectively. These results (Figure 9, Figure 10, Figure 11 and Figure 12) indicated that the proposed device can function well under fabrication tolerance errors of the Gap and the SW width.

An oscillated behavior is obtained for crosstalk and insertion loss in Figure 9, Figure 10, Figure 11 and Figure 12. This is because the crosstalk and insertion loss results are correlated to the coupling transfer energy between two closer SW segment units (affected directly by the coupling loss) that also have an oscillated behavior (Figure 4b).

The tolerance ranges data of the width SW (±45 nm) and the Gap (±20 nm) can be used in future work to fabricated this design using a high accuracy fabrication process that is in use today, with an accurate range of ±20 nm shift.

To show the advances of using slot-waveguide technology a comparison was done between the visible light demultiplexer devices that are commonly used today and have been previously published and the proposed device in this work as shown in Table 4. Table 4 compares the key characteristics of the demultiplexer which are as follows: number of channels, dimensions (length × width), max insertion loss (IL), best crosstalk (CT), and the year of publication.

By analyzing the results shown in Table 4 it is clear that the proposed design has several advantages compared to the other demultiplexer devices which are small dimensions, low insertion loss, and more channels. The ability to obtain more channels is because SW technology can support light guiding in both polarization modes TE and TM which can be utilized in a VLC system for increasing data bit rate using the WDM technique.

## 4. Conclusions

To conclude, this work demonstrates a new and novel design of an ultra-compact 1 × 4 green/red light wavelength demultiplexer based on a multi SW structure under TM/TE polarization mode.

This study of the light coupling between six segment SWs and the oscillation characteristics between them under both mode polarization TE and TM has shown the ability to obtain a lower coupling length size compared to the classical waveguide or SW using MMI structures. This ability has been utilized to control the light switching between two closer segment SWs and to obtain an ultra-compact device.

Results have shown that the eight operating wavelengths range from 530–560 nm to 630–660 nm with an accurate spacing of 10 nm. The overall length of the demultiplexer is only 104.5 µm with excellent crosstalk values between (−21.14) and (−25.63) dB, low power loss values between 0.1 and 0.138 dB, and large optical bandwidth values between 8.98 and 9.39 nm.

These values indicate that demultiplexer can be useful, especially, for increasing bit rate data in networking applications that applied WDM technology in the red/green light spectrum.

This work can be expanded to a vast number of wavelength dividers, e.g., 1 × 8, 1 × 16, and 1 × 32 and can be implemented on a different range of operating wavelengths such as C-band and other bands. The functionality of this device shows the great potential of using a multi SW technology to increase performances in VLC systems.

## Figures and Tables

**Table 1:** Refractive index as a function of green (TM mode) and red (TE mode) wavelengths.

**Wavelength (nm)**	**530**	**540**	**550**	**560**
n_Silica_	1.46	1.46	1.459	1.458
n_GaN_	2.424	2.419	2.414	2.41
**Wavelength (nm)**	**630**	**640**	**650**	**660**
n_Silica_	1.457	1.456	1.456	1.456
n_GaN_	2.385	2.382	2.38	2.377

**Table 2:** Coupling length as a function of green and red wavelengths.

**Wavelength (nm)**	**530**	**540**	**550**	**560**
Coupling length (µm)	3.21	3.07	2.83	2.65
**Wavelength (nm)**	**630**	**640**	**650**	**660**
Coupling length (µm)	2.08	2.01	1.99	1.88

**Table 3:** Values of crosstalk losses and bandwidth.

**Wavelength (nm)**	**530**	**540**	**550**	**560**
Port number	3	2	4	1
Crosstalk (dB)	−21.14	−22.54	−23.1	−24.05
Loss (dB)	0.134	0.113	0.123	0.117
Bandwidth (nm)	8.98	9.05	9.15	9.39
**Wavelength (nm)**	**630**	**640**	**650**	**660**
Port number	4	2	3	1
Crosstalk (dB)	−22.3	−22.61	−25.63	−24.1
Loss (dB)	0.136	0.138	0.1	0.124
Bandwidth (nm)	9.01	9.08	9.13	9.29

**Table 4:** Comparison between key properties of virus demultiplexer technology types [3,28].

Demultiplexer Technology Type	Number of Channels	Size (Length × Width) (mm)	Max IL (dB)	Best CT (dB)	Year
Holographic concave grating reflector (1200 lines/mm)	3	30 × 35	2	20	2005
Prism	3	79 × 94	12	6.8	2008
RGB demux based on silicon-nitride multi core photonic crystal fiber	3	5.5 × 1	2.3	20	2018
RGB demux based on polycarbonate multi core polymer optical fiber	3	20 × 1	1.3	19	2019
A Four Demultiplexer Green/Red Light Using Multi Slot Waveguide Structures	8	0.1045 × 0.0031	0.12	24.1	In this work
